# Clinical Value Analysis of Hepatectomy Based on Minimally Invasive Surgical Imaging for Hepatolithiasis

**DOI:** 10.1155/2022/3306771

**Published:** 2022-08-31

**Authors:** Shubin Zhang, Zhongqiang Xing, Xinbo Zhou, Zixuan Hu, Jianhua Liu

**Affiliations:** Department of General Surgery, The Second Hospital of Hebei Medical University, Shijiangzhuang 050000, Hebei, China

## Abstract

**Objective:**

To investigate the clinical value of hepatectomy based on minimally invasive surgical images in the treatment of hepatolithiasis.

**Methods:**

The clinical data of 87 patients with hepatolithiasis who received treatment in the Department of General Surgery of our hospital from February 2020 to September 2021 were retrospectively analyzed. According to different surgical methods, the patients were divided into minimally invasive group (*n* = 43) and laparotomy group (*n* = 44). Perioperative conditions and stone clearance rate were compared.

**Results:**

The preoperative conditions of patients in the two groups were comparable, and the average operation time in the minimally invasive group was significantly longer than that in the laparotomy group (*t* = 18.783,*P* < 0.001). There was no significant difference in intraoperative bleeding, postoperative fasting time, postoperative complications, and stone clearance between the two groups (*P* > 0.05). Postoperative hospital stay was significantly lower in the minimally invasive group than that in the laparotomy group (*t* = −0.486,*P* < 0.001).

**Conclusion:**

Hepatectomy based on minimally invasive surgical imaging for hepatolithiasis is safe and feasible, has high clinical value, and can achieve similar short-term clinical efficacy to laparotomy and reduce the postoperative hospital stay of patients, reflecting its minimally invasive advantages, and it is worthy of clinical application.

## 1. Introduction

Hepatolithiasis is a common biliary tract disease in China, and the incidence of hepatolithiasis accounts for 16% to 18% of cholelithiasis [1]. The treatment is relatively complex, and the residual rate of stones and the incidence of complications after surgery are relatively high. Hepatectomy is still the preferred treatment [2]. At present, there are laparotomy and minimally invasive surgery (endoscopy). The choice of surgery needs to be based on the preoperative auxiliary examination and diagnosis. Traditional laparotomy is invasive and risky. In recent years, with the rapid development of endoscopic surgical techniques in biliary surgery, the advantages of minimally invasive surgical imaging in the treatment of hepatolithiasis have been gradually highlighted and gradually applied in clinical practice [3]. However, due to the complexity and extensiveness of the distribution of hepatolithiasis, minimally invasive surgery still lacks detailed criteria in terms of preoperative planning, ultrasound navigation, hemobilia and biliary stricture treatment, and postoperative management, and the safety and effectiveness need to be studied in depth. Therefore, this study investigated the clinical value of hepatectomy based on minimally invasive surgical images in the treatment of hepatolithiasis in order to provide a reference for the surgical treatment of intrahepatic and extrahepatic bile duct stones.

### 1.1. Study Subjects and Methods

#### 1.1.1. Study Subjects

The clinical data of 87 patients with hepatolithiasis who received diagnosis and treatment in the Department of General Surgery of our hospital from February 2020 to September 2021 were retrospectively analyzed. They were divided into minimally invasive surgery group and laparotomy group according to surgical methods. There were 43 patients in the minimally invasive group and 44 patients in the laparotomy group.

Inclusion criteria were as follows: (1) hepatolithiasis diagnosed by imaging examination; (2) hepatectomy; (3) complete clinical data; and (4) agreed to sign informed consent.

Exclusion criteria were as follows: (1) combined with other serious systemic diseases; (2) combined with suppurative cholecystitis, gangrenous cholecystitis, and acute pancreatitis; and (3) coagulation disorders.

The study was approved by the ethics committee of The Second Hospital of Hebei Medical University (No.hbmu021), and the patients signed the informed consent.

## 2. Methods

### 2.1. Preoperative Preparation

All patients underwent preoperative examinations such as enhanced CT, MRCP, chest radiograph, electrocardiogram, and blood routine before operation. Preoperation education and adaptive exercise shall be carried out for patients to prepare their gastrointestinal tract. All patients took Shugan Jianpi Rongshi decoction. The compound includes Bupleurum chinense, Paeonia lactiflora, tangerine peel, Pinellia ternata, Codonopsis pilosula, Poria cocos, Atractylodes macrocephala, Glycyrrhiza, Magnolia officinalis, qianqiancao, haijinsha, Chuanpo stone, chenneijin, Burmese wormwood, and neem.

### 2.2. Surgical Methods

Open hepatectomy: the patient was under general anesthesia, and a vertical incision with a length of about 15cm was made on the right side of rectus abdominis. The scope of resection was determined according to the patient's condition. The blood flow of hepatic portal was blocked by the same method as that of laparoscopy. The diseased liver was severed by clamp method. The wound was covered with hemostatic sponge and greater omentum, and a drainage tube was placed.

Minimally invasive hepatectomy operation steps refer to Guidelines for Minimally Invasive Surgical Treatment of Hepatolithiasis (Edition 2019). Preoperative ultrasonography, choledochoscopy, CT scan, and arteriography were performed to determine the extent of liver resection margin. General anesthesia was performed with endotracheal intubation. During the resection, the liver segment and lobe could be used as units. The lesion could be removed by minimally invasive surgery. The diseased liver was divided by the clamping method. Adequate hemostasis was performed. The wound surface was washed. The diseased liver lobe and the pipeline in the Glisson fiber sheath of the liver were dissected. The harmonic scalpel could be used to achieve hemostatic effect and relieve pneumoperitoneum for the small pipeline in the liver.

### 2.3. Outcome Measures

Patients' clinical data were collected as follows: gender, age, BMI, Child–Pugh classification, smoking status, history of diabetes, and history of hypertension. The operation time, intraoperative blood loss, intraoperative abdominal drainage volume, postoperative hospital stay, drainage tube placement time, postoperative incision infection, bile leakage, pulmonary infection and other complications, and postoperative stone clearance rate were observed in the two groups. The calculation formula of stone clearance rate is as follows: the number of cases without residual stones in postoperative imaging and choledochoscopy in the minimally invasive surgery group or open surgery group/the total number of cases in the laparoscopic surgery group or open surgery group.

### 2.4. Data Analysis

SPSS 24.0 software was used to statistically describe and analyze the data. When quantitative data conformed to normal distribution, they were described as mean ± standard deviation, and enumeration data were expressed as rate. Measurement data of patients in the two groups were compared by the *t*-test, and enumeration data were analyzed by *χ*^2^ or Fisher exact probability test. *P* values represent 2-sided probabilities, with *P* < 0.05 considered statistically different.

## 3. Results

### 3.1. Baseline Data

The comparison of preoperative general data showed that there was no significant difference in age, gender, body mass index, Child–Pugh classification, and past medical history between the two groups (*P* > 0.05), as shown in Table 1.

### 3.2. Comparison of Intraoperative Conditions

The differences in the operation time, intraoperative blood loss, and intraoperative laparoscopic drainage volume between the laparoscope group and laparotomy group had statistical significance (*P* < 0.05). There was no significant statistical difference in the proportion of T-tube drainage placement (*P* > 0.05), as shown in Table 2.

### 3.3. Comparison of Postoperative Conditions

Patients in the minimally invasive group had shorter postoperative hospital stays and drainage tube placement days than the laparotomy group. Postoperative complications occurred in 4 cases in the minimally invasive group and 8 cases in the laparotomy group, and the difference had no different significance between the two groups. The difference in stone clearance rate between the two groups was not significant (*P* > 0.05), as shown in Table 3.

## 4. Discussion

The most effective treatment for patients with hepatobiliary calculi is partial hepatectomy, which can remove the stones, remove the narrow area of the bile duct and the atrophic liver parenchyma, thereby reducing the risk of stone recurrence [4]. Open hepatectomy is effective but invasive to patients. Minimally invasive surgical image-based hepatectomy for hepatolithiasis is minimally invasive, and there were many reports [5]. There is still a lack of sufficient evidence to support its advantages in terms of safety and efficacy compared with open hepatectomy. The results of this study showed that minimally invasive hepatectomy was comparable to open hepatectomy in removing stones and had significant advantages in reducing operation time, intraoperative blood loss, intraoperative abdominal drainage volume, and postoperative complications and shortening hospital stay.

Intraoperative bleeding control is a difficult point in minimally invasive hepatectomy. Perfect imaging data, reasonable hepatic blood flow occlusion, liver transection and hemostatic devices, control of central venous pressure, and skilled microscopic operation are considered the key to control intraoperative blood loss. Patients with hepatolithiasis tend to have varying degrees of inflammatory adhesions in the diseased liver segment, which can become difficult to perform laparoscopically [6], and hemostatic techniques used for open hepatectomy such as vascular ligation and compression hemostasis are equal to laparoscopic inability to flexibly use. In this study, intraoperative blood loss in the minimally invasive group was not significantly higher than that in the laparotomy group, which was consistent with the results of Ye et al. [7]. This result may be due to the high-resolution magnification of the surgical field provided by laparoscopy and the more meticulous dissection of the liver parenchyma using a laparoscopic multifunctional surgical dissector. Hepatobiliary surgeons can combine preoperative imaging data and mark the hepatic resection line under the guidance of ultrasound to monitor the separation of blood vessels and bile ducts in real time. It may also be related to the maturity of hepatobiliary surgeons' concept awareness, proficiency in operation techniques, and continuous improvement of laparoscopic surgical instruments. It is inconsistent with the research results of Li et al. [8] and Wang et al. [9]. The results show that the amount of intraoperative bleeding in the minimally invasive surgery group is less than that in the open surgery group, and the difference between the two groups is statistically significant.

In this study, the operation time of the minimally invasive group was longer than that of the laparotomy group. It was considered that the main reason was not only that laparoscopic surgery required hepatectomy but also that laparoscopic bile duct exploration, stone extraction, liver section bile duct suture, and other operations were completed, and the operation steps were complicated compared with open left hepatectomy; in addition, bile duct inflammation caused by long-term bile duct stones led to adhesion of the first porta hepatis to the surrounding tissues, increasing the difficulty of laparoscopic anatomical separation of the first porta hepatis, resulting in prolonged operation time.

n this study, the postoperative hospital stay of the minimally invasive group was shorter than that of the laparotomy group. Considering that the laparoscopic technique has less irritation to organs and tissues around the liver, the abdominal wall surgical incision is reduced, the incision infection rate is reduced, and with the surgeon 's laparoscopic technique becoming more skilled, the postoperative hospital stay of the patients is shortened. The total incidence rate of complications in the minimally invasive group was lower than that in the laparotomy group, but the difference between the two groups had no significant difference. Considering that the sample size was small, it had a certain impact on the statistical results.

Stone clearance rate is an important indicator to evaluate the effect of hepatectomy in the treatment of hepatolithiasis. In this study, the stone clearance rate was 88.4% in the minimally invasive group and 88.6% in the laparotomy group, and there was no significant difference between the two groups. In accordance with relevant domestic and foreign studies [3, 10], it was reported that there was no significant difference in stone clearance rate between the minimally invasive group and the laparotomy group. It may be related to the routine application of ultrasonography and choledochoscopy in minimally invasive surgery to detect stones deep in the liver parenchyma and identify the location, size, and extent of stones and the location of biliary strictures.

In summary, laparoscopic hepatectomy for left hepatolithiasis is safe and feasible and has high clinical value. It can achieve similar short-term clinical efficacy to laparotomy, reflecting the advantages of minimally invasive surgery. It is superior to laparotomy in controlling intraoperative bleeding and shortening postoperative hospital stay. However, because this study is a single center retrospective study, in order to provide a higher level of evidence-based medical evidence support for the safety and effectiveness of laparoscopic hepatectomy in the treatment of hepatolithiasis, a prospective randomized controlled trial with a sufficient number of cases is also needed.

## Figures and Tables

**Table 1:** Baseline characteristics.

Characteristics	Minimally invasive group (*n* = 43)	Laparotomy group (*n* = 44)	t/*χ*^2^	*P*
Age	53.19 ± 2.37	53.89 ± 2.33	−1.387^*∗*^	0.169

Gender			0.335	0.563
Male	15 (34.9%)	18 (65.1%)		
Female	28 (40.9%)	26 (59.1%)		

Body mass index	21.50 ± 0.71	22.00 ± 0.82	−0.730^*∗*^	0.506

Child–Pugh classification			0.859	0.354
Grade A	39 (90.07%)	37 (84.1%)		
Grade B	4 (9.3%)	7 (15.9%)		

Smoking history			0.186	0.666
Yes	10 (23.3%)	12 (27.3%)		
No	33 (76.7%)	32 (72.7%)		

Diabetes			0.132	0.716
Yes	6 (14%)	5 (11.4%)		
No	37 (86%)	38 (88.6%)		

Hypertension			0.055	0.814
Yes	7 (16.3%)	8 (18.2%)		
No	36 (83.7%)	36 (81.8%)		

*Note.* ^*∗*^refers to the use of *t*-test.

**Table 2:** Comparison of intraoperative conditions.

	Minimally invasive group (*n* = 43)	Laparotomy group (*n* = 44)	t/*χ*^2^	*P*
T-tube drainage	36 (83.7%)	32 (72.7%)	1.540	0.215
Procedure time	214.60 ± 13.78	139.82 ± 0.75	18.783^*∗*^	<0.001
Intraoperative bleeding	201.44 ± 8.37	200.09 ± 1.04	0.536^*∗*^	0.599
Intraoperative abdominal drainage	146.63 ± 0.74	187.11 ± 4.29	−26.281	<0.001

*Note.* ^*∗*^refers to the use of *t*-test.

**Table 3:** Comparison of postoperative conditions.

	Minimally invasive group (*n* = 43)	Laparotomy group (*n* = 44)	*t*/*χ*^2^	*P*
Postoperative hospital stay	6.71 ± 0.95	9.09 ± 1.04	−4.864^*∗*^	<0.001
Postoperative fasting time	1.43 ± 0.79	1.89 ± 0.79	−1.165^*∗*^	0.263
Drainage tube placement days	3.56 ± 0.73	5.56 ± 0.73	−5.840^*∗*^	<0.001
Postoperative complications	4 (9.3%)	8 (18.2%)	1.442	0.230
Incision infection	1	2		
Biliary fistula	2	3		
Lung infection	1	3		
Stone clearance			0.001	0.969
Yes	38 (88.4%)	39 (88.6%)		
No	5 (11.6%)	5 (11.4%)		

*Note.* ^*∗*^refers to the use of *t*-test.

## Data Availability

The data used to support the findings of this study are available from the corresponding author upon request.
